# Genome structure and molecular phylogeny of the only Eurasian *Boechera* species, *Boechera falcata* (Brassicaceae)

**DOI:** 10.1093/g3journal/jkaf117

**Published:** 2025-06-20

**Authors:** Danil Zilov, Terezie Mandáková, Marina Popova, Martin A Lysak, Michael D Windham, Vladimir Brukhin

**Affiliations:** Wellcome Sanger Institute, Wellcome Genome Campus, Hinxton, Cambridge CB10 1SA, UK; ITMO University, ChemBio Cluster Lomonosova 9, Saint Petersburg 197101, Russia; Central European Institute of Technology, Masaryk University, Kamenice 753/5, Brno 625 00, Czech Republic; Faculty of Science, Institute of Experimental Biology, Masaryk University, Kamenice 753/5, Brno 625 00, Czech Republic; The Skolkovo Institute of Science and Technology, Bolshoy Blvd 30-1, Moscow 121205, Russia; Central European Institute of Technology, Masaryk University, Kamenice 753/5, Brno 625 00, Czech Republic; Faculty of Science, Institute of Experimental Biology, Masaryk University, Kamenice 753/5, Brno 625 00, Czech Republic; Department of Biology, Duke University, Biological Sciences Bldg., Durham, NC 27708, USA; Department of Molecular Biology and Biotechnology, School of Natural and Environmental Sciences, Newcastle University, Newcastle upon Tyne NE1 7RU, UK; Lab of Embryology and Reproductive Biology, Komarov Botanical Institute, RAS, 2 Prof. Popov Str., St. Petersburg 197376, Russia

**Keywords:** *Boechera falcata*, Brassicaceae, genome structure, chromosome-level genome assembly and annotation, chromosome rearrangements, comparative chromosome painting, molecular phylogeny, chloroplast genome

## Abstract

*Boechera falcata* (Turcz.) Al-Shehbaz, previously known as *Arabis turczaninowii* Ledeb., is a herbaceous perennial of the East Siberian, boreal-steppe ecotype. It is the sole species of the diverse genus *Boechera* found on the Eurasian continent, with all other species endemic to North America and Greenland. Likely migrating from North America to Eastern Siberia via the Bering Land Bridge during the Pleistocene glaciation, *B. falcata* presents a unique case for genomic study. The genus *Boechera* is notable for its many allodiploid and triploid apomicts, which have arisen through complex hybridization of sexual species and ecotypes. To date, only the genomes of 2 American *Boechera* species, *B. stricta* and *B. retrofracta*, have been sequenced and analyzed. In this study, we sequenced, assembled to the chromosome level, and analyzed the highly homozygous 189.36 Mb genome of *B. falcata* (2*n* = 14). Molecular phylogenetic analysis of nuclear and organelle genomes revealed a high degree of relatedness to North American relatives. Cytogenetic analysis identified all 22 genomic blocks of crucifers, showing that 5 of the 7 *B. falcata* chromosomes are collinear with their ancestral counterparts, while 2 have undergone inversions. Allelic analysis of the apomixis marker *APOLLO* gene revealed that *B. falcata* contains only sex alleles. The availability of the *B. falcata* genome will advance studies of the evolution and phylogeny of Brassicaceae species and the mechanisms of apomixis, providing a crucial resource for future research in plant genetics and breeding.

## Introduction

The genus *Boechera* (Á. Löve & D. Löve Brassicaceae) comprises ∼75 sexual diploid taxa and around 355 genetically distinct hybrid lineages, predominantly distributed in western North America (Alexander *et al*. 2013; Hay *et al*. 2023). *Boechera falcata* (Turcz.) Al-Shehbaz, previously known as *Arabis turczaninowii* Ledeb., is the sole species of this genus found in Eurasia, specifically endemic to Eastern Siberia and the Far East. Molecular marker data suggest that its origin may be linked to the migration of ancestral forms from North America to Siberia via the Bering Land Bridge (Al-Shehbaz 2005; Windham and Al-Shehbaz 2007; Kiefer *et al*. 2009; Alexander *et al*. 2013; Brukhin *et al*. 2019).

Earlier classifications placed members of *Boechera* within the genus *Arabis* based on superficially similar morphological traits. However, karyological studies revealed chromosomal differences, prompting Löve and Löve (1976) to separate the genus *Arabis* (s.l.) into New World species with *x* = 7 (now classified as *Boechera*) and Old World species with *x* = 8 (retained in *Arabis*). Subsequent molecular phylogenetic analyses have confirmed that these 2 genera belong to distinct clades within the Brassicaceae (Koch *et al* 1999, 2003; Beilstein *et al*. 2010; Nikolov *et al*. 2019; Hendriks *et al*. 2023), demonstrating that their similarities are convergent rather than indicative of a close evolutionary relationship.

Species of the genus *Boechera* are closely related to the well-studied model plant *Arabidopsis thaliana* within the Brassicaceae family. *Boechera* is unique within this family for its documented apomixis (Böcher 1951, 1969; Brukhin *et al*. 2019). Apomixis is a form of asexual reproduction through seeds, where the embryo arises as a genetic clone of the maternal plant, developing parthenogenetically without the fusion of male and female germ cells. This ability to produce clones of a maternal plant and hence fix the desired genotypes in subsequent generations has significant potential for agricultural applications, facilitating effective plant breeding strategies (Grossniklaus *et al*. 2002; Bicknell and Koltunow 2004; Van Dijk *et al*. 2009; Brukhin 2017).

iDploid and triploid apomictic accessions of *Boechera* exhibit signs of hybridogenic origin, leading changes in chromosome structure such as alloploidy, ascending dysploidy, substitution of homeologous chromosomes, and the appearance of aberrant extra chromosomes (Mandáková *et al*. 2015; Brukhin *et al*. 2019). Therefore, species from the genus *Boechera* serve as unique models for molecular genetic studies of apomixis and the influence of hybridization of sexual forms on its occurrence.

A locus correlated with female apomeiosis, the *APOmixis Linked Locus* (*APOLLO*), has been identified, encoding an Asp-Glu-Asp-Asp-His exonuclease. Its expression is downregulated in sexual ovules as they enter meiosis and upregulated in apomeiotic ovules (Corral *et al*. 2013; Bakin *et al*. 2022). *APOLLO* exhibits biallelic inheritance with both “apo-” and “sex-” alleles, differing by a 20-nucleotide polymorphism in the 5′ untranslated region of the gene. All previously tested apomictic *Boechera* accessions were heterozygous for *APOLLO* alleles, having at least 1 apo-allele and 1 sex-allele, while all sexual accessions were homozygous for sex-alleles (Corral *et al*. 2013; Kliver *et al*. 2018; Brukhin *et al*. 2019; Bakin *et al*. 2022). A high correlation (98.4%) was observed between the presence of *APOLLO* loci associated with apomixis and the apomictic mode of reproduction. The function of *APOLLO* during reproduction in *Boechera* remains unclear (Brukhin *et al*. 2019). Kliver *et al*. (2018) found 2 additional, more distant copies of *APOLLO*, which may indicate past duplication events. It was shown that the apo- and sex-alleles of *APOLLO* arose after the separation of the genus *Boechera*, forming 2 distinct clades. An evolutionary scenario was proposed in which one of the *APOLLO* copies could have acquired a new function in the common ancestor of *Boechera*, leading to the separation of apomictic lineages. According to the Ka/Ks ratio = 1.4646 of *APOLLO* alleles suggests that the branch leading to the apo-alleles is under positive selection, typical for paralogs that have acquired a new function (Kliver *et al*. 2018 ; Brukhin *et al*. 2019).

To date, whole-genome assemblies have been published for only 2 *Boechera* species: *B. stricta* (Lee *et al*. 2017) and *B. retrofracta* (Kliver *et al*. 2018). These species are of particular interest because, while they reproduce predominantly sexually, many of their hybrids are apomicts. The Far Eastern *B. falcata* studied here is a key outgroup species for understanding the genome structure of the genus and the evolution of genetic loci associated with apomixis. This study aims to: (1) Achieve a chromosome-scale genome assembly of *B. falcata*. (2) Perform a phylogenetic analysis to explore its relationship with North American *Boechera* species. (3) Analyze genome structure, perform Hi-C genomic analysis, comparative genome analysis, and phylogenetic analysis based on nuclear and chloroplast DNA. (4) Study of the chromosome structure and evolution using comparative chromosome painting. (5) Study alleles of the apomixis-associated *APOLLO* locus (encoding NEN exonuclease) in *B. falcata* and add to the phylogenetic tree of *APOLLO* locus isoforms of the *Boechera* species published earlier (Kliver *et al*. 2018).

## Material and methods

### Plant material

**Fig. 1.**
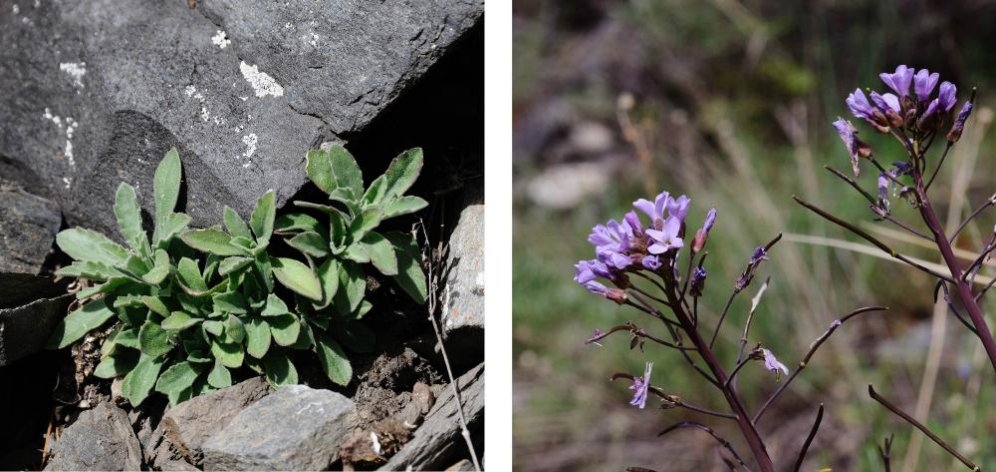
Plants of *B. falcata* (leave rosette and blooming plants with siliques) growing in the Kolyma River valley in the Magadan region, from which seeds were collected.

Seeds of *B. falcata* were collected from plants (Fig. 1) in natural habitats: a petrophytic open community of *Artemisia gmelinii* Weber ex Stechm. and *B. falcata* on the southern slope with a steepness of 45° (Tenkinsky district of the Magadan region, 10 km southeast of the village of Orotuk—62° 01′ 50.586″ N, 148° 38′ 46.591″ E).

Plants were cultivated from seeds in the greenhouse of CEITEC Masaryk University under the standard conditions (16/8 h light/dark, 21/18°C, 15 µmol/m^2^/s). Specimens are deposited at the Herbarium of Masaryk University (BRNU): BRNU sheet numbers: 696,364 (https://brnu.jacq.org/BRNU696264) and 696,267 (https://brnu.jacq.org/BRNU696267) (Supplementary Fig. 1).

### Microsatellite genotyping

Genomic DNA was extracted using the Qiagen DNeasy Plant Mini Kit. Microsatellite allele variation was assessed at 15 previously published loci (A1, A3, B6, Bdru266, BF3, BF9, BF11, BF15, BF18, BF19, BF20, C8, E9, ICE3, and ICE14) following Beck *et al*. (2012). Forward primers for each locus were labeled with either 6-FAM or HEX. Multiplex polymerase chain reaction (PCR) was performed for sets of 3 loci simultaneously. Each 50 μl reaction contained 15 μl of 2× Qiagen Multiplex PCR Master Mix, 3 μM of the primer set, and ∼40 ng of gDNA template. The PCR conditions included an initial denaturation at 95°C for 15 min, followed by 30 cycles of denaturation at 95°C for 30 s, annealing at 53°C for 90 s, and extension at 72°C for 60 s, with a final extension step at 60°C for 30 min. PCR products were first analyzed on a 1–3% agarose gel. Capillary electrophoresis was then performed by Macrogen using a 400 HD standard. Electropherograms were analyzed with the Microsatellite plugin of Geneious software, and species identity was determined using the Boechera Microsatellite Website https://windhamlab.biology.duke.edu/ (Li *et al*. 2017).

### Chromosome preparation and comparative chromosome painting

Mitotic and meiotic (pachytene) chromosome preparations were performed according to the protocol outlined by Mandáková and Lysak (2016). Before analysis, suitable slides were pretreated with RNase (100 mg/ml) and pepsin (0.1 mg/ml). Comparative chromosome painting was performed according to Mandáková *et al*. (2020). Fluorescent signals were examined and captured using a Zeiss Axioimager epifluorescence microscope equipped with a CoolCube camera (MetaSystems). Images were acquired separately for each of the 4 fluorochromes using their respective excitation and emission filters (AHF Analysentechnik). The collected images were then pseudocolored and merged using Adobe Photoshop CS6 (Adobe Systems).

### Whole-genome sequencing strategy

#### DNA extraction and prob preparation

DNA was extracted following Huang *et al*. (2023) protocol. The *B. falcata* genome was sequenced on the 3 WGS platforms: Illumina NovaSeq with 150 bp PE sequencing run, final 75 Gb data obtained, with 243× coverage; Pacbio sequel II (HiFi/CCS mode/cell) (1 SMRT cell), genome coverage—129×; Hi-C genomic analysis technique ∼50 M Read-Pairs, genome coverage—30×.

#### Raw data filtration and preprocessing

Raw Illumina reads and HiC reads trimming were processed using Trimmomatic v0.32 (Bolger *et al*. 2014) with the following parameters ILLUMINACLIP:2:30:10:2:True LEADING:3 TRAILING:3 MINLEN:36. HiFi reads evaluation of length and quality stats was performed with pauvre tool (https://github.com/conchoecia/pauvre). The quality assessment of HiFi reads using the Pauvre tool indicated a mean read length of 13,387 bases and an N50 value of 13,975 bases. The Phred quality scores ranged from 20 to 50, with a peak at 40 (Supplementary Fig. 2).

#### Genome coverage estimation

Estimation of the genome coverage based on the 23-mer distribution and *k*-mer based statistics was performed using thegenomic k-mer counter Meryl (https://github.com/marbl/meryl), k-mer databases of HiFi and Illumina WGS reads, and GenomeScope2 (Ranallo-Benavidez *et al*. 2020).

### Genome assembly and quality control

For genome assembly, we performed a bake off with 4 different assemblers: for HiFi reads: flye v2.9.2-b1786 (Kolmogorov *et al*. 2019), verkko v1.4.1 (Rautiainen *et al*. 2023), nextdenovo 2.5.2 (Hu *et al*. 2024 ), and hifiasm v0.19.6-r595 (Cheng *et al*. 2021) (Table 1). Flye was run with the –keep-haplotypes option, while verkko and nextdenovo targeted a genome size of 200 Mbp. Nextdenovo and hifiasm were executed with standard parameters for HiFi reads. The assembly process was automated using the hifiblender pipeline (https://github.com/zilov/hifiblender).

**Table 1.** Comparative statistics of genome assembly by different assemblers.

	NextDenovo	Flye	Hifiasm	Verkko
Contigs no.	50	145	1,232	2,041
Total length (bp)	189,363,209	201,258,247	249,420,951	246,125,585
GC (%)	35.95	35.96	36.53	37.85
N50	12,407,843	6,341,302	30,472,172	7,370,569
L50	7	11	4	11
A k-mers not found in reads (missing)	1,929	2,859	21,778	75,086
A k-mers overly represented in assembly	1,460,638	3,908,427	31,538,183	27,968,096
Merqury QV	62.83	62.09	46.84	47.36
Merfin QV*	32.99	30.69	24.05	23.71
Total low-coverage regions:	0	0	0	0
BUSCO	C: 98.8% [S: 95.8%, D: 3.0%], F: 0.2%, M: 1.0%, n: 4,596	C: 98.8% [S: 95.7%, D: 3.1%], F: 0.2%, M: 1.0%, n: 4,596	C: 98.8% [S: 95.9%, D: 2.9%], F: 0.2%, M: 1.0%, n: 4,596	C: 98.7% [S: 95.6%, D: 3.1%], F: 0.2%, M: 1.1%, n: 4,596

Quality of the assembly was assessed using multiple metrics: N50, L50, BUSCO completeness, and QV (Quality Value). QUAST (Gurevich *et al*. 2013) was utilized for contiguity statistics, BUSCO (Seppey *et al*. 2019) for completeness assessment, and Merfin (Formenti *et al*. 2022) for QV calculation using a Meryl HiFi + Illumina hybrid k-mer database (*k* = 23). Coverage of the genome was calculated with GenomeScope2 (Ranallo-Benavidez *et al*. 2020).

Haplotype phasing was performed using hapdup (https://github.com/KolmogorovLab/hapdup) with default parameters. Polishing was conducted with nextpolish (Hu *et al*. 2020) using Illumina reads, with the number of steps (3 for the first pseudo-haplotype and 2 for the second) determined by QV value progression checked on hybrid HiFi and Illumina Meryl database.

Scaffolding and genome curation Hi-C was facilitated by yahs (Zhou *et al*. 2023), followed by genome curation using juicebox (Robinson *et al*. 2018). The hic-scaffolder pipeline (https://github.com/zilov/hic-scaffolder) streamlined Hi-C read alignment and scaffolding processes. Finally, we aligned the orientation and order of chromosomes to match the *Boechera stricta* assembly (NCBI GenBank assembly ID: GCA_018361395.1).

### Organelle DNA assembly and annotation

Mitochondrial and chloroplast genomes were assembled using contigs from the whole-genome assembly after polishing and HiC scaffolding steps. For the mitochondrial genome, we employed MitoHiFi (Uliano-Silva *et al*. 2023), using the *B. stricta* mitochondrion as a reference to identify and assemble a complete circular mitochondrial sequence.

The chloroplast genome assembly was more complex. MitoHiFi was unable to resolve a circular molecule, so we used a manual approach. The *B. stricta* chloroplast genome served as a reference for identifying relevant contigs. We then performed a BLAST search to find intersections between these contigs. The chloroplast genome assembly required additional manual steps. While MitoHiFi software typically assembles circular organelle genomes, it failed to automatically reconstruct the complete circular chloroplast genome in our case. Therefore, we developed an alternative approach. First, we used the *B. stricta* chloroplast genome as a reference to identify chloroplast-derived contigs. Then, we performed BLAST analysis to find overlapping regions between these contigs. Using these overlapping sequences, we manually constructed the complete circular chloroplast genome. Chloroplast and mitochondrial genome annotation and visualization were performed using GeSeq (Tillich *et al*. 2017) and manually curated by OGDRAW (Greiner *et al*. 2019), respectively.

### Nuclear genome annotation

To call for repetitive elements in the genome, we used EarlGrey (Baril *et al*. 2024) repeat masking pipeline. A custom repeat database was created by combining the PlantRep database (Luo *et al*. 2022) and the nrTEplants dataset (Contreras-Moreira *et al*. 2021). Initial masking was conducted with the “eukarya” search term. De novo repeat identification performed with RepeatModeler (Flynn *et al*. 2020). The custom database was then used for subsequent rounds of masking to ensure identification of repetitive elements specific to plant genomes. EarlGrey utilizes a combination of tools, including RepeatModeler (Flynn *et al*. 2020), RepeatMasker (https://repeatmasker.org/), LTR_FINDER (Xu and Wang 2007), and others to refine and cluster repeat sequences. For coding gene annotation, we employed BRAKER v3.0.8 (Gabriel *et al*. 2024) in compleasm (Huang and Li, 2023) mode, utilizing the Viridiplantae OrthoDB (Kriventseva *et al*. 2019) protein database for homology-based prediction.

### Comparative analysis of *B. falcata* and *B. stricta* genomes

We compared the assembled genome of *B. falcata* with the published *B. stricta* genome (NCBI GenBank assembly ID: GCA_018361395.1). Both genomes were evaluated using QUAST and BUSCO. QV calculations were performed differently: for *B. falcata*, k-mer analysis was carried out based on the hybrid HiFi and Illumina reads (https://github.com/arangrhie/T2T-Polish/tree/master/merqury), while for *B. stricta* k-mer analysis performed using only Nanopore reads due to their availability. Repeat masking and annotation were performed on both genomes. Synteny analysis between *B. falcata* and *B. stricta* chromosomes was conducted using SyRI (Goel *et al*. 2019). Whole-genome alignment visualization was generated with the help of D-GENIES (Cabanettes and Klopp 2018) to produce a comparative dotplot.

### Phylogenetic analysis of *B. falcata* among the other *Boechera* and Brassicaceae species

The phylogenetic analysis of *B. falcata* was conducted in the context of other *Boechera* and Brassicaceae species using a combination of Angiosperms353 and Brassicaceae764 probe sets, following a methodology similar to that described by Hay *et al*. (2023). Loci were assembled using HybPiper version 2.1.7 with Illumina reads. Assembled loci were added to the existing locus files of other *Boechera* species. Each locus was aligned using MAFFT (Katoh and Toh 2008) with the L-INS-I method. Alignments were concatenated into a supermatrix using FASconCAT-G (Kück and Longo 2014) and subsequently trimmed using trimAl (Capella-Gutiérrez *et al*. 2009) with the -automated1 setting to reduce gaps and missing data. Phylogenetic tree construction was performed using RAxML-NG v1.2.0 (Kozlov *et al*. 2019), employing the GTR + F + R model and generating 1,000 bootstrap replicates.

To analyze the phylogeny based on the chloroplast genome, we extracted 47 chloroplasts genes (listed in Supplementary Table 1) from the complete chloroplast assemblies of *Boechera* species and other Brassicaceae species reported elsewhere (Zhu *et al*. 2021). Phylogenetic analysis of chloroplast genomes was performed in MEGA (v.11.0.10): ClustalW(Codons) option with default settings was used for alignment of gene sequences. Phylogenetic tree was constructed with the Neighbor-Joining test with bootstrap 1,000.

### Analysis of *APOLLO* sex- and apo-alleles in *B. falcata* Genome

The *APOLLO* gene (Corral *et al*. 2013) was identified in the annotated genome using BLAST search. Additional gene sequences for phylogenetic analysis were obtained from Bakin *et al*. (2022). Multiple sequence alignment was performed using MUSCLE (Edgar 2004). Phylogenetic tree construction was carried out with RAxML-NG v1.2.0 (Kozlov *et al*. 2019), employing the following parameters: -all -model JTT + G –bs-trees 1,000 –tree pars{10}.

### Visualization

Visualization of HiC-plot was performed with cooler (https://github.com/open2c/cooler), with each point on the plot representing 80kbp. Circos plot was built with pyCirclize (https://github.com/moshi4/pyCirclize). The phylogenetic trees were visualized with iTol (Letunic and Bork 2021). The synteny plots were built with plotsr (Goel and Schneeberger 2022), dotplot of genome-to-genome alignment with dgenies (Cabanettes and Klopp 2018).

## Results and discussion

### Chromosome count

**Fig. 2.**
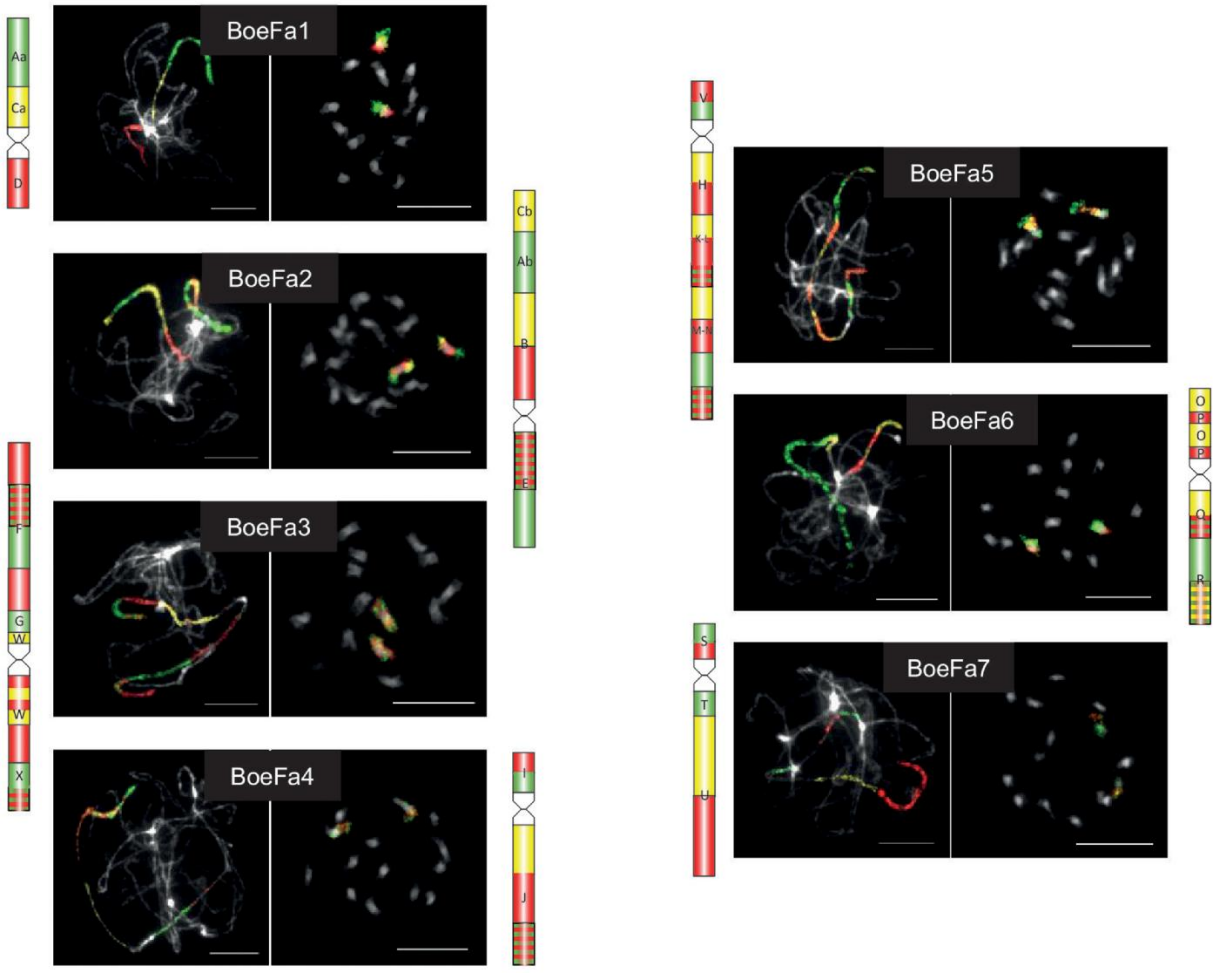
Karyotype structure of *B. falcata* inferred from comparative chromosome painting (CCP). The 7 chromosomes (BoeFa1–BoeFa7) were visualized through CCP using *Arabidopsis* BAC contigs as painting probes applied to pachytene, mitotic, and diakinesis chromosome spreads. Painting signals are shown in experimental colors, reflecting the fluorochrome(s) used to label specific genomic blocks (GBs). All chromosomes were counterstained with DAPI and presented as grayscale images. Scale bars, 10 µm.

Chromosome number was determined from young anthers of 10 plants cultivated from seeds, confirmed by microsatellite genotyping as *B. falcata*. All plants were confirmed to be diploid, with a chromosome count of 2*n* = 14 (Fig. 2).

### Karyotype structure

**Fig. 3.**
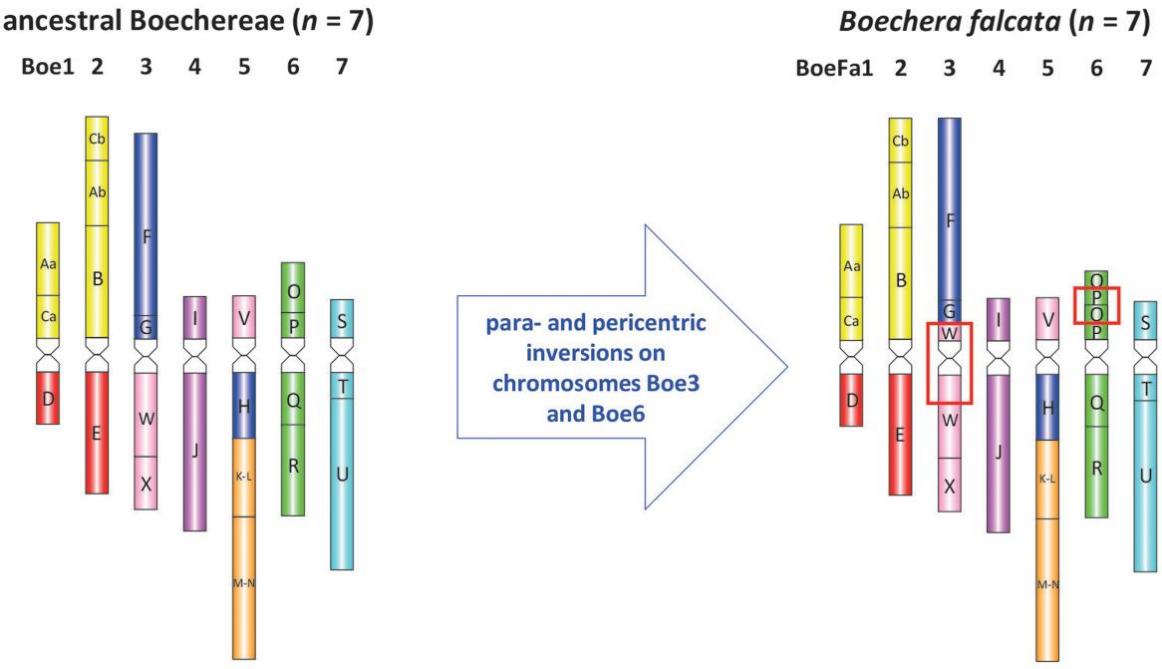
Chromosome evolution of *B. falcata* from the ancestral Boechereae genome (Mandáková *et al*. 2020). Color coding of 22 genomic blocks (GBs, A to X) reflects their position on the 8 ancestral chromosomes in the Ancestral Crucifer Karyotype (Lysak *et al*. 2016). The ideograms are drawn to scale, whereby the size of GBs corresponds to the size of homeologous blocks in the *A. thaliana* genome (The Arabidopsis Information Resource, TAIR; http://www.arabidopsis.org).

Bacterial artificial chromosome-based comparative chromosome painting was employed to investigate the genome structure of *B. falcata*. Probes were arranged according to the ancestral Boechereae genome structure (Mandáková *et al*. 2020), allowing the mapping of all 22 crucifer genomic blocks (Mandáková *et al*. 2019) onto *B. falcata* chromosomes (BoeFa1–BoeFa7) (Figs. 2 and  3). Notably, 5 of the 7 *B. falcata* chromosomes (BoeFa1, BoeFa2, BoeFa4, BoeFa5, and BoeFa7) exhibit collinearity with their corresponding chromosomes in the ancestral Boechereae genome (Mandáková *et al*. 2020). In contrast, BoeFa3 contains a pericentric inversion involving genomic block W, while BoeFa6 undergoes a paracentric inversion within its short arm, with breakpoints located within genomic blocks O and P (Figs. 2 and 3).

### k-mer based statistics indicates a high level of *B. falcata* genome homozygosity

**Fig. 4.**
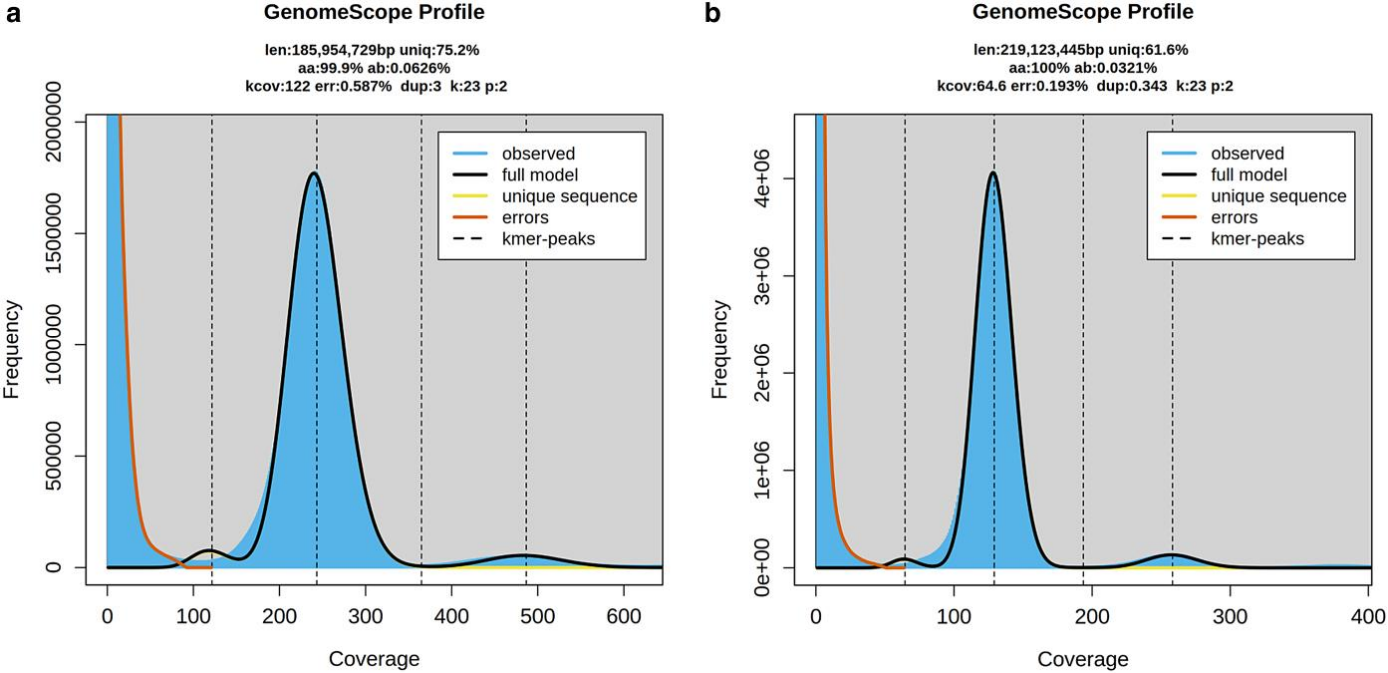
Distribution of 23-mers for detection genome size and evaluation of homo/heterozygosity (a) for Illumina PE reads and (b) HiFi libraries. Only 1 major peak is present in both Illumina (at 249× coverage) and HiFi (129×) libraries.

The *k*-mer spectrum built by GenomeScope2 software (Ranallo-Benavidez *et al*. 2020) for both Illumina and HiFi reads is shown in Fig. 4. The 23-mer distribution has 1 major peak at 249× coverage corresponding to diploid 23-mers (shared between homologous chromosomes), also small heterozygous peaks (122× and 64×) were detected (Fig. 4). According to the assessment, the genome was highly homozygous with 99.9% of AA k-mers for both Illumina and HiFi reads. The genome size of *B. falcata* was estimated to be close to 185 Mbp by Illumina k-mers and 219 Mbp by HiFi k-mers, which is in the range of the previous estimations of a minimal genome size of ∼200 Mbp in *Boechera* (Anderson *et al*. 2011). Homozygosity of genomes in *Boechera* is characteristic of sexual reproduction, since all apomicts studied are usually heterozygous, allodiploids or polyploids (Brukhin *et al*. 2019). Sexuality in *B. falcata* was also confirmed by cytoembryological studies (Vinogradova *et al*. 2023).

### Assessment of the quality of genome assembly performed by different assemblers

To identify the most efficient and correct *B. falcata* genome construction, we compared 4 different assemblers: NextDenovo, flye, hifiasm, and verkko (Table 1). The assemblies varied considerably in their contiguity and quality metrics. NextDenovo assembler demonstrated the highest contiguity with only 50 contigs achieving N50 of 12,407,843 bp, L50 of 7. Evaluation of the assembly completeness was performed using BUSCO, complete BUSCO score is 98.8% (95.8% single-copy, 3.0% duplicated). This assembly also exhibited the highest Merqury QV (62.83) and Merfin QV* (32.99) scores, indicating greater accuracy compared to the other assemblies.

The hifiasm assembler produced the most contigs (1232) and achieved the highest N50 value of 30,472,172 bp. However, it showed a higher number of overrepresented k-mers and lower QV scores compared to the NextDenovo and flye assemblers.

Indeed, all assemblies showed complete BUSCO genes (≥98.7%), indicating high assembly completeness under different assembly strategies. The GC content was similar in all assemblies and ranged from 35.95 to 37.85%. However, based on the obtained metrics, we chose the NextDenovo assembler for further haplotype phasing, polishing, analysis, and improvement of the Hi-C scaffolding due to its favorable balance of continuity, completeness, and accuracy.

### Genome polishing

After splitting haplotypes to dual assembly with hapdup, both primary and alternate haplotype underwent 3 rounds of polishing using Illumina reads (Table 2). Each polishing step progressively improved the assembly quality, as evidenced by the decreasing number of missed k-mers and increasing Merqury QV scores. After the final polishing step, the primary haplotype achieved a Merqury QV of 68.04, while the alternative haplotype reached 67.6, indicating high accuracy in both haplotypes.

**Table 2.** Genome polishing information.

Polishing step	Primary haplotype missed k-mers/Merqury QV	Alternative haplotype missed k-mers/Merqury QV
Step 1	893/66.88	1,020/66.3
Step 2	837/67.16	732/67.74
Step 3	683/68.04	757/67.6

### Hi-C scaffolding and chromosome-level assembly

**Fig. 5.**
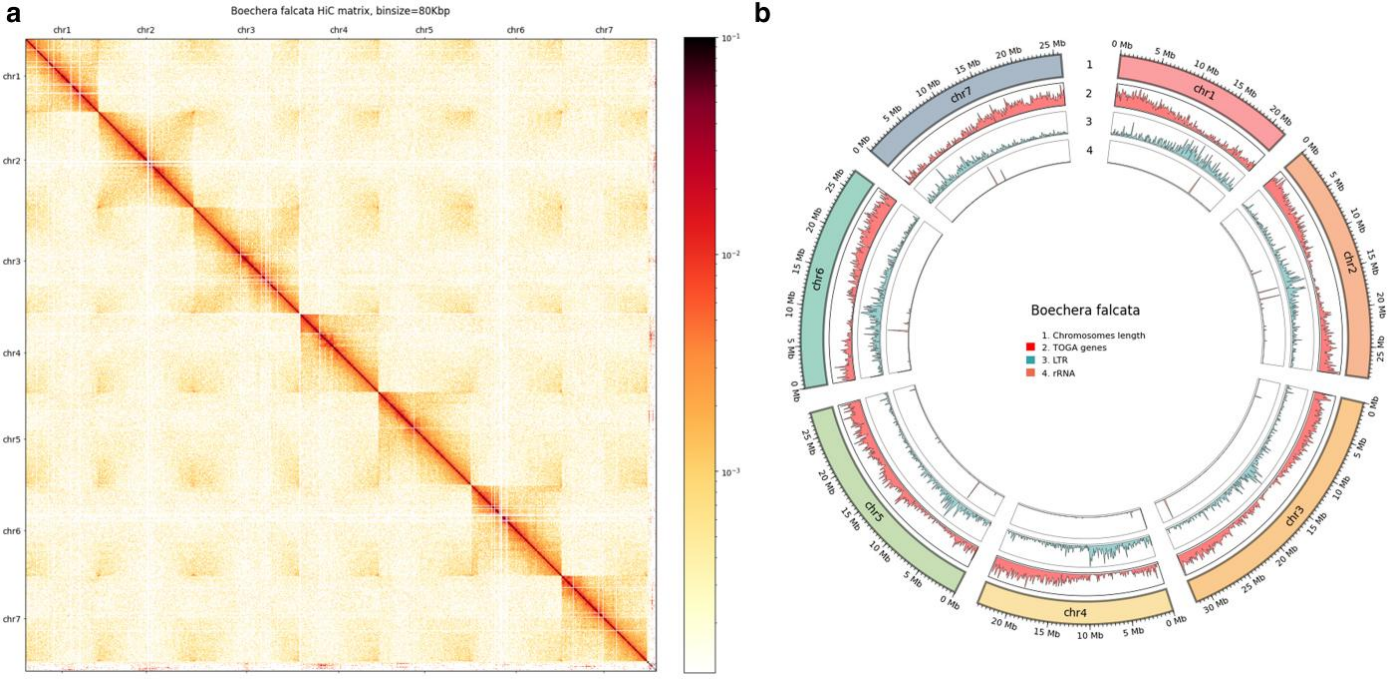
a) Hi-C contact matrix of *B. falcata* genome at 80 kbp resolution. The 7 distinct chromosomes are clearly visible along the diagonal, indicating successful chromosome-level assembly. The intensity of red coloration represents the frequency of interactions between genomic regions, with darker red indicating higher interaction frequencies. b) Circos plot of the genome assembly. HiC map was built with 80 kbp bin size, circos plot includes (1) lengths of chromosomes, (2) frequency of genes in 100 kbp bin size, (3) frequency of long tandem repeats in 100 kbp bin size, and (4) frequency of rRNA genes in the same bin size.

Following genome assembly, haplotype phasing, and polishing, we performed Hi-C scaffolding to achieve a chromosome-level assembly. This process resulted in the successful construction of 7 chromosomes, consistent with the known karyotype of *Boechera* species (Fig. 5). The final assembly contained a total of 32 gaps. The assemblies of the primary and alternate haplotypes are available on the NCBI, BioProjects: PRJNA1077252 and PRJNA1100512, respectively.

### Analysis of repetitive elements

The repeat masking analysis revealed that 38.46% of the genome is made up of repetitive elements (Table 3). Long terminal repeat (LTR) retrotransposons were the most common class of repeats, accounting for 19.76% of the genome with 24,870 instances identified. DNA transposons represented the second most prevalent class, masking 6.23% of the genome. Long interspersed nuclear elements (LINEs) and short interspersed nuclear elements (SINEs) accounted for 2.39 and 0.008% of the genome, respectively. Other repetitive elements, including simple repeats, microsatellites, and RNA genes, comprised for a total of 0.65% of the genome. Additionally, 8.82% of the masked regions were classified as uncharacterized repetitive elements.

**Table 3.** Repeats revealed by RepeatMasker.

Class of repetitive element	Total length	*n* of elements	% of genome
DNA	11,783,246	17,307	6.235
LINE	4,527,617	8,007	2.39
LTR	37,373,250	24,870	19.76
Other (simple repeat, microsatellite, RNA)	1,221,953	2,131	0.65
Penelope	24,530	259	0.01
Rolling circle	1,123,003	2,785	0.59
SINE	15,061	234	0.01
Unclassified	16,681,735	47,524	8.82
Total	72,750,395	103,117	38.46

### Prediction of protein-coding genes

Homology-based prediction yielded a total of 27,516 protein-coding genes predicted. BUSCO analysis demonstrated completeness of annotation, with 99.2% of the 4,596 BUSCO groups present and completes (Table 4).

**Table 4.** Homology-based gene prediction in *B. falcata* genome.

Genes	27,516
Exons	161,159
Introns	130,644
mRNAs	30,516
BUSCOs	C: 99.2% [S: 78.83%, D: 20.37%] F: 0.13%, M: 0.67%, N: 4,596

### Comparative analysis of *B. falcata* and *B. stricta* genomes

Comparative analysis of the *B. falcata* and *B. stricta* genomes revealed similarities in overall structure, as well as notable differences in assembly quality (Table 5). Before analysis, the *B. falcata* genome was oriented with the *B. stricta* genome. Both species possess 7 chromosomes and demonstrated highly comparable genome sizes of 189.36 and 190.54 Mb and exhibited similar assembly contiguity metrics N50 of 27.20 and 26.95 Mb for *B. falcata* and *B. stricta* correspondingly. BUSCO genome completeness scores are nearly identical for both species 98.8 and 98.7% for *B. falcata* and *B. stricta* accordingly, as well as gene counts, 27,516 in *B. falcata*, 27,538 in *B. stricta*. However, *B. falcata* demonstrated higher assembly accuracy with higher Merqury QV (68.04 vs 27.49) and Merfin QV (33.08 vs 23.32) scores. Both genomes were assembled to chromosome level as diploid assemblies. Thus, combination of high structural similarity and improved accuracy of the *B. falcata* genome assembly provides the basis for a detailed comparative analysis of its genome with other species of the genus *Boechera*.

**Table 5.** Comparison of genome characteristics of *B. falcata* and *B. stricta.*

Metric	*B. falcata*	*B. stricta*
Genome length (bp)	189,363,209	190,536,481
Chromosomes (x)	7	7
N50 (bp)	27,200,968	26,945,205
BUSCO genome	C: 98.8% [S: 95.8%, D: 3.0%], F: 0.2%, M: 1.0%, *n*: 4,596	C: 98.7% [S: 95.7%, D: 3.0%], F: 0.3%, M: 1.0%, *n*: 4,596
Merqury QV	68.04	27.49
Merfin QV	33.08	23.32
Genes	27,516	27,538
BUSCO annotation	C: 99.2% [S: 78.83%, D: 20.37%] F: 0.13%, M: 0.67%, N: 4,596	C: 98.98% [S: 79.29%, D: 19.69%] F: 0.13%, M: 0.89%, N: 4,596

**Fig. 6.**
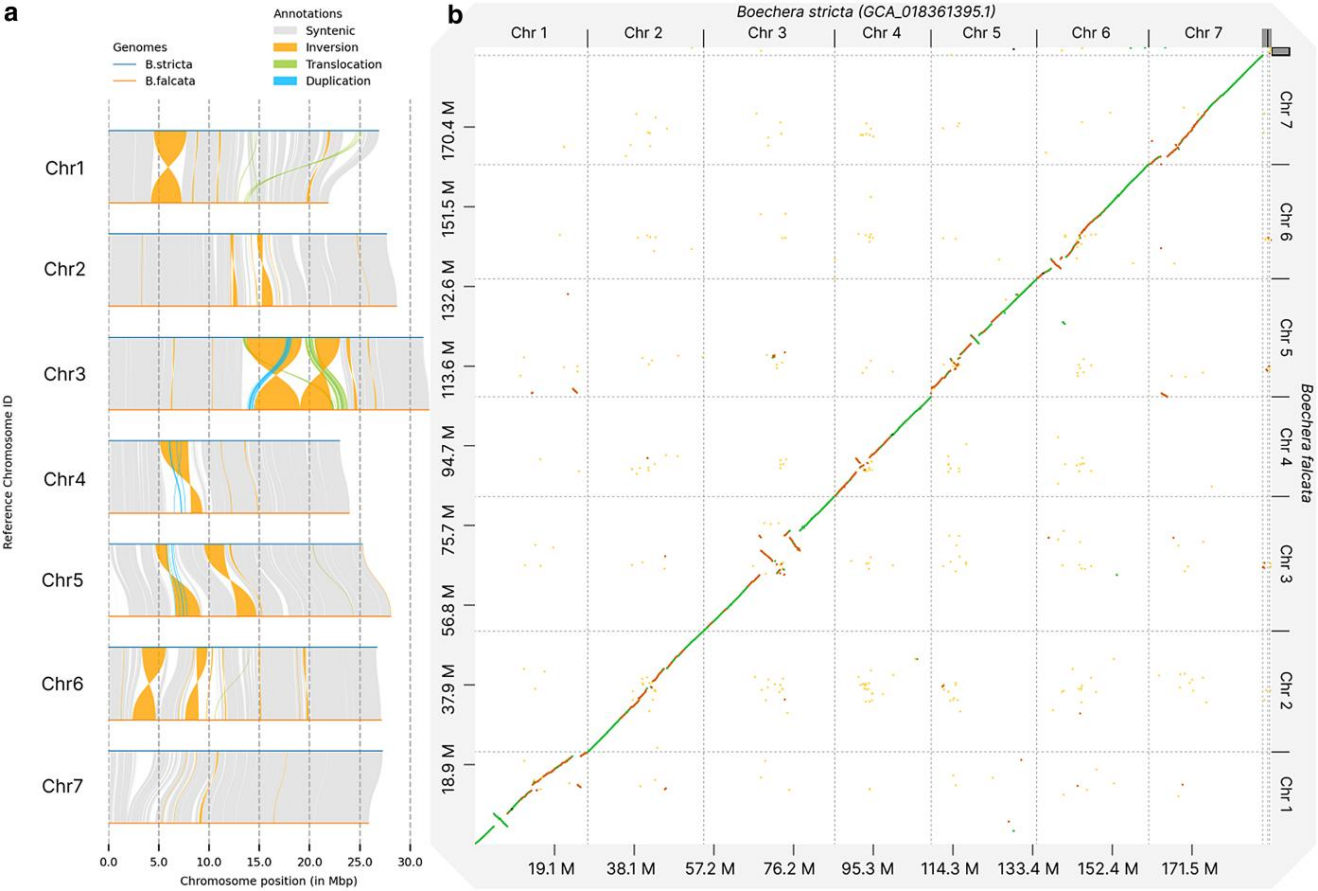
Illustration of the structural variations between *B. stricta* and *B. falcata* genomes. a) Chromosomal rearrangements, each chromosome of *B. falcata* is aligned to *B. stricta*. The orange regions represent inversions. Gray areas indicate syntenic regions. b) Scatter plot of genome-wide comparison: chromosomes of *B. stricta* on the x-axis and *B. falcata* on the y-axis. Diagonal green lines represent syntenic regions with high sequence identity (>70%), and yellow regions indicate areas with lower sequence identity (<70%). Prominent large-scale inversions are visible, especially in chromosomes 1, 3, and 5 in (a) and corresponding off-diagonal patterns in (b). The dot plot highlights the overall conservation of chromosomal structure between the 2 species and also highlights significant inversions and areas of differing sequence identity across the genomes.

Analysis of structural variations between the *B. falcata* and *B. stricta* genomes revealed significant genomic rearrangements (Fig. 6). The comparison identified 294 syntenic regions, covering the majority of both genomes (Table 6). A total of 66 inversions were detected, spanning ∼23.67 Mb in the reference genome (*B. stricta*) and 23.60 Mb in the query genome (*B. falcata*). These inversions represent substantial chromosomal rearrangements among the 2 species. Large regions of both genomes were found to be nonsyntenic: 19.24 Mb are unique to *B. stricta* and 18.36 Mb are unique to *B. falcata*, indicating species-specific genomic content. Figure 6 shows these structural variations on synteny plot (a) and dotplot (b).

**Table 6.** Synteny analysis characteristics.

Variation_type	Count	Length_ref (*B. stricta*)	Length_qry (*B. falcata*)
Syntenic regions	294	141,724,185	141,383,384
Inversions	66	23,669,784	23,602,144
Translocations	348	3,730,041	3,629,426
Duplications (*B. stricta*)	150	476,406	—
Duplications (*B. falcata*)	359	—	1,366,155
Not aligned (*B. stricta*)	801	19,235,167	—
Not aligned (*B. falcata*)	1,030	—	18,357,653

### *B. falcta* falls into the homozygous sex allele clade on the *APOLLO* phylogenetic tree

The *APOLLO* gene, encoding the NEN3 exonuclease, is an important apomixis-associated gene in *Boechera* (Corral *et al*. 2013; Brukhin *et al*. 2019). The *APOLLO* locus has previously been shown to have multiple alleles (Corral *et al*. 2013; Kliver *et al*. 2018). All studied apomictic plants carry at least one of the “apo” alleles, and the sexual accessions are homozygous for the “sex” alleles.

**Fig. 7.**
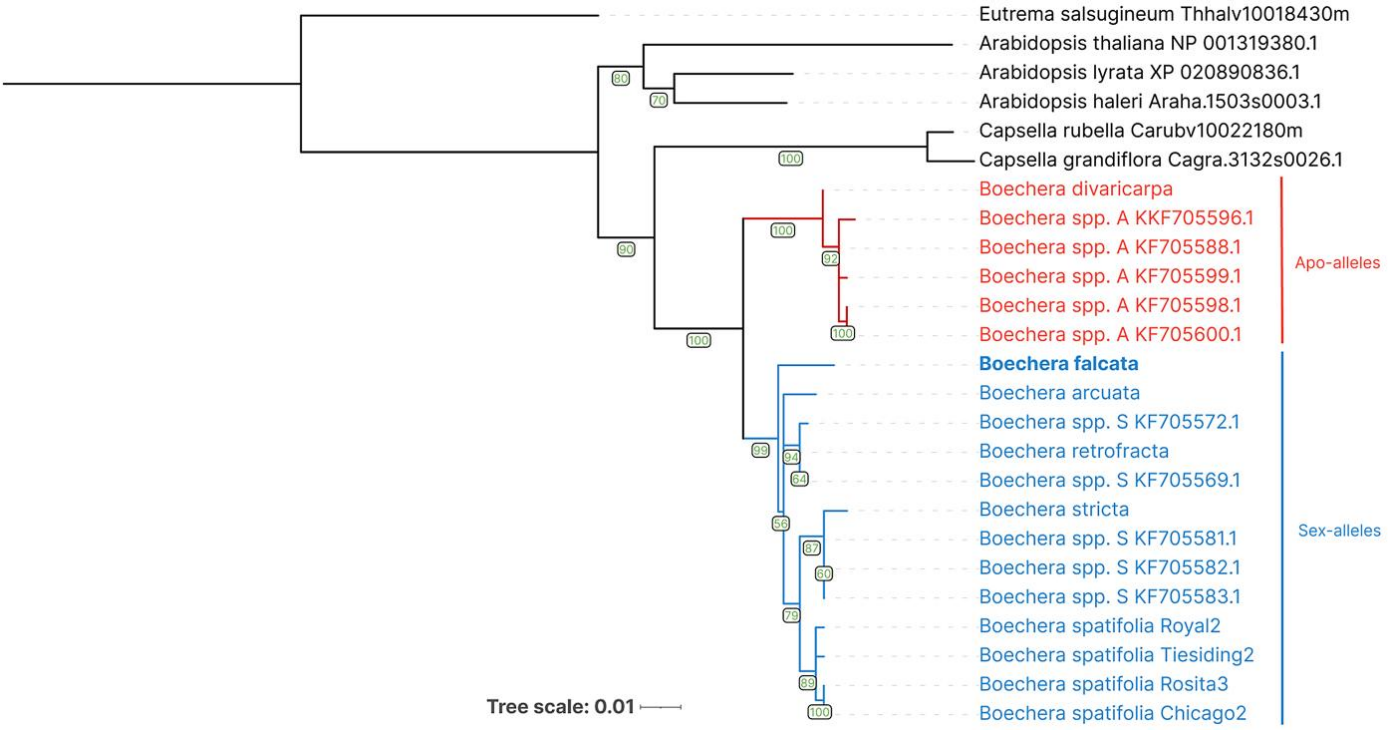
Phylogenetic tree of *APOLLO* gene alleles in *Boechera* and related species. Sequences of Eutrema *salsugineum*, *A. thaliana*, *A. lyrata*, *A. haleri*, *Capsella rubella*, and *C. grandiflora* were used as outgroups. The 2 main clades within *Boechera* are highlighted: apomictic species in red and sexual species in blue including *B. falcata*. The numbers in the nodes represent bootstrap support values.

We decided to further explore the *APOLLO* locus in the *B. falcata* genome. The phylogenetic analysis of the *APOLLO* gene revealed a clear division between 2 groups of *Boechera* species, in accordance with their mode of reproduction (Fig. 7), as was previously described (Kliver *et al*. 2018). The tree shows 2 distinct clades within the *Boechera* genus, with strong bootstrap support (100) for the split between these groups. One clade comprises apomictic *Boechera* spp., including apomictic *Boechera* hybrid M4B, whose genome we assembled to chromosome scale previously (NCBI BioProject PRJNA774175), which forms a well-supported monophyletic group. The other clade comprises *B. falcata*, *B. arcuata*, *B. retrofracta*, *B. stricta*, and several *Boechera* spp., along with multiple *B. spatifolia* accessions, which reproduce sexually. Notably, *B. falcata* is positioned within this second clade, clustering closely with sexual *B. arcuata* and *Boechera spp.* (KF705572.1). This study demonstrates clear phylogenetic differentiation of the *APOLLO* gene between the 2 groups of *Boechera* species, with the *B. falcata* group included in the clade that along with *B. stricta* and other known sexual species.

### Chloroplast and mitochondrial genome assembly and annotation

**Fig. 8.**
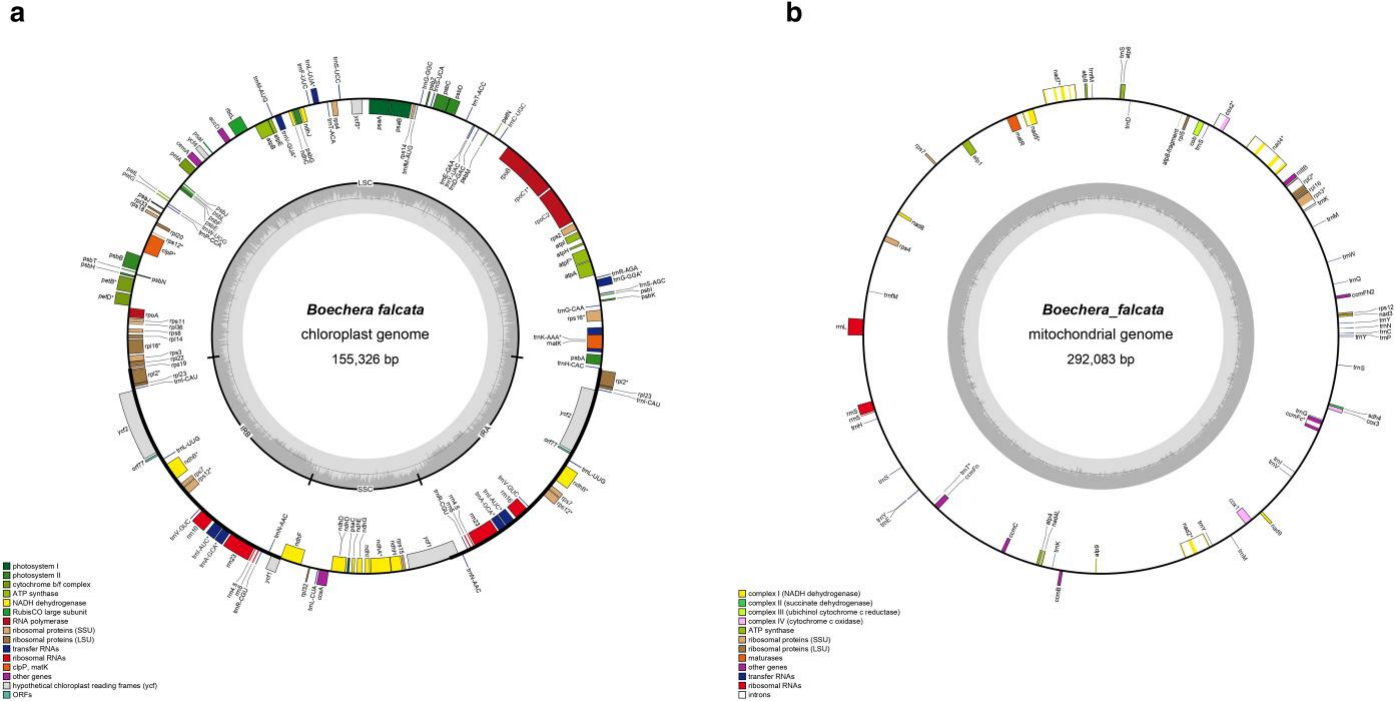
Circular representation of *B. falcata* chloroplast (a) and mitochondrial (b) genome. Gray circle inside is GC% content.

To facilitate further evolutionary relationship study of nonmodel *Boechera* species, phylogenetic research, and species identifications, we performed *B. falcata* chloroplast and mitochondrial genomes assembly and annotation (Fig. 8). The entire size of the chloroplast genome is 155,326 bp. 79 unique CDS, 30 tRNAs and 4 rRNA were identified in the chloroplast genome. Large single-copy (LSC) part and small single-copy (SSC) part of the chloroplast DNA occupy 84,428 and 17,952 bp, respectively. GC contents is 36.41%. All these indicators are consistent with those in the chloroplast genomes of other *Boechera* species as shown in Table 7.

**Table 7.** Comparison of several *Boechera* species chloroplast genomes.

Species name	RefSeq accession number	Genome size (bp)	LSC Length (bp)	SSC Length (bp)	IR Length (bp)	Number of genes			GC (%)			
						CDS	tRNAs	rRNAs	Total genome	LSC	SSC	IR
*B. falcata*	Chloroplast	155,326	84,428	17,952	26,473	79	30	4	36.41	34.20	29.42	42.33
*Boechera suffrutescens*	NC_049600.1	155,114	84,285	17,915	26,457	79	30	4	36.43	34.18	29.52	42.36
*B. stricta*	NC_049599.1	155,033	84,237	17,884	26,456	79	30	4	36.39	34.15	29.38	42.33
*Boechera pulchra*	NC_049598.1	155,348	84,483	17,951	26,457	79	30	4	36.42	34.17	29.49	42.37
*Boechera puberula*	NC_049597.1	155,011	84,189	17,928	26,447	79	30	4	36.48	34.26	29.63	42.35
*Boechera laevigata*	NC_049596.1	155,083	84,266	17,891	26,463	79	30	4	36.45	34.22	29.48	42.33
*Boechera davidsonii*	NC_049594.1	154,935	84,107	17,914	26,457	79	30	4	36.46	34.24	29.57	42.33
*Boechera breweri*	NC_049593.1	155,147	84,347	17,886	26,457	79	30	4	36.44	34.21	29.56	42.33

Length of the mitochondrial DNA in *B. falcata* is 292,083 bp and GC content is 44.90% (Fig. 8). Comparative data on the mitochondrial genome size and GC content in *B. falcata*, *B. stricta*, and *A. thaliana* are shown in Table 8. Table 9 indicates the type and functions of the genes identified in mitochondrial DNA of *B. falcata*, *B. stricta*, and *A. thaliana.* All in all, 33 protein-coding genes, 17 tRNAs and 3 rRNA were annotated in the mitochondrial genome of *B. falcata*.

**Table 8.** Comparative data on mitochondrial genome size and GC content in *B. falcata*, *B. stricta*, and *A. thaliana*.

Species name	RefSeq accession number	Genome size (bp)	GC content %
*B. falcata*	Mitochondrion	292,083	44.90
*B. stricta*	NC_042143.1	271,601	44.93
*A. thaliana*	NC_037304.1	367,808	44.79

**Table 9.** Characteristics of the genes identified in mitochondrial DNA of *B. falcata, B. stricta*, and *A. thaliana.*

Group of genes	Name of genes		
	*B. falcata*	*B. stricta*	*A. thaliana*
ATP synthase	atp1, atp4, atp6, atp8, atp9	atp1, atp4, atp6, atp8, atp9	atp1, atp4, atp6, atp8, atp9
Cytochrome c biogenesis	ccmB, ccmC, ccmFc, ccmFn, ccmFN2	ccmB, ccmC, ccmFc, ccmFn	ccmB, ccmC, ccmFC, ccmFN1, ccmFN2
Ubiquinol cytochrome c reductase	Cob	Cob	cob
Cytochrome c oxidase	cox1, cox2, cox3	cox1, cox2, cox3	cox1, cox2, cox3
Maturases	matR	matR	matR
Transport membrane protein	mttB	mttB	mttB
NADH dehydrogenase	nad1, nad2, nad3, nad4, nad4L, nad5, nad6, nad7, nad9	nad1, nad2, nad3, nad4, nad4L, nad5, nad6, nad7, nad9	nad1, nad2, nad3, nad4, nad4L, nad5, nad6, nad7, nad9
Large subunit of ribosome	rpl2, rpl5, rpl16	rpl2, rpl5, rpl16	rpl2, rpl5, rpl16
Small subunit of ribosome	rps3, rps4, rps7, rps12	rps3, rps4, rps12	rps3, rps4, rps7, rps12
Succinate dehydrogenase	sdh4	sdh4	—
Ribosomal RNAs	rrn5, rrn18, rrn26	rrn5, rrn18, rrn26	rrn5, rrn18, rrn26
Transfer RNAs	trnC, trnD, trnE, trnG, trnH, trnI, trnK, trnM, trnN, trnP, trnQ, trnS, trnT, trnV, trnW, trnY, trnfM	trnC, trnD, trnE, trnG, trnH, trnI, trnK, trnM, trnN, trnP, trnQ, trnS, trnW, trnY, trnfM	trnC, trnD, trnE, trnG, trnH, trnI, trnK, trnM, trnN, trnP, trnQ, trnS, trnW, trnY, trnfM

### Phylogeny of *B. falcata* and its relationship with other *Boechera* and Brassicaceae species

A phylogenetic tree of Brassicaceae constructed based on 47 chloroplast genes (Supplementary Fig. 3, *B. falcata* highlighted in bold) confirms the monophyly of the sampled *Boechera* species within the tribe Boechereae. Notably, *B. falcata*, the first representative of the genus from Eurasia to be included in such a chloroplast genome analysis, is deeply nested within the single major *Boechera* clade recovered, which otherwise consists entirely of North American species.

The placement of *B. falcata* firmly within the clade of predominantly North American *Boechera* species, rather than in a basal position or as a sister group to North American taxa, suggests a North American origin for *B. falcata.* The fact that this Eurasian species falls within the only identified *Boechera* lineage, rather than forming a distinct Eurasian clade, further supports this interpretation. This phylogenetic position implies that *B. falcata* maternal lineage originated in North America and subsequently dispersed to the North–East of Eurasia, likely across Beringia during periods when a land bridge was present, or possibly via long-distance dispersal. This finding aligns with previous phylogeographic studies based on fewer chloroplast markers, which also indicated a North American center of diversity for *Boechera* (Dobeš *et al.* 2004a, 2004b; Kiefer *et al*. 2009). This chloroplast-based phylogeny provides evidence for *Boechera* intrageneric intercontinental exchange, which is an example of East Asian/North American floristic disjunction.

**Fig. 9.**
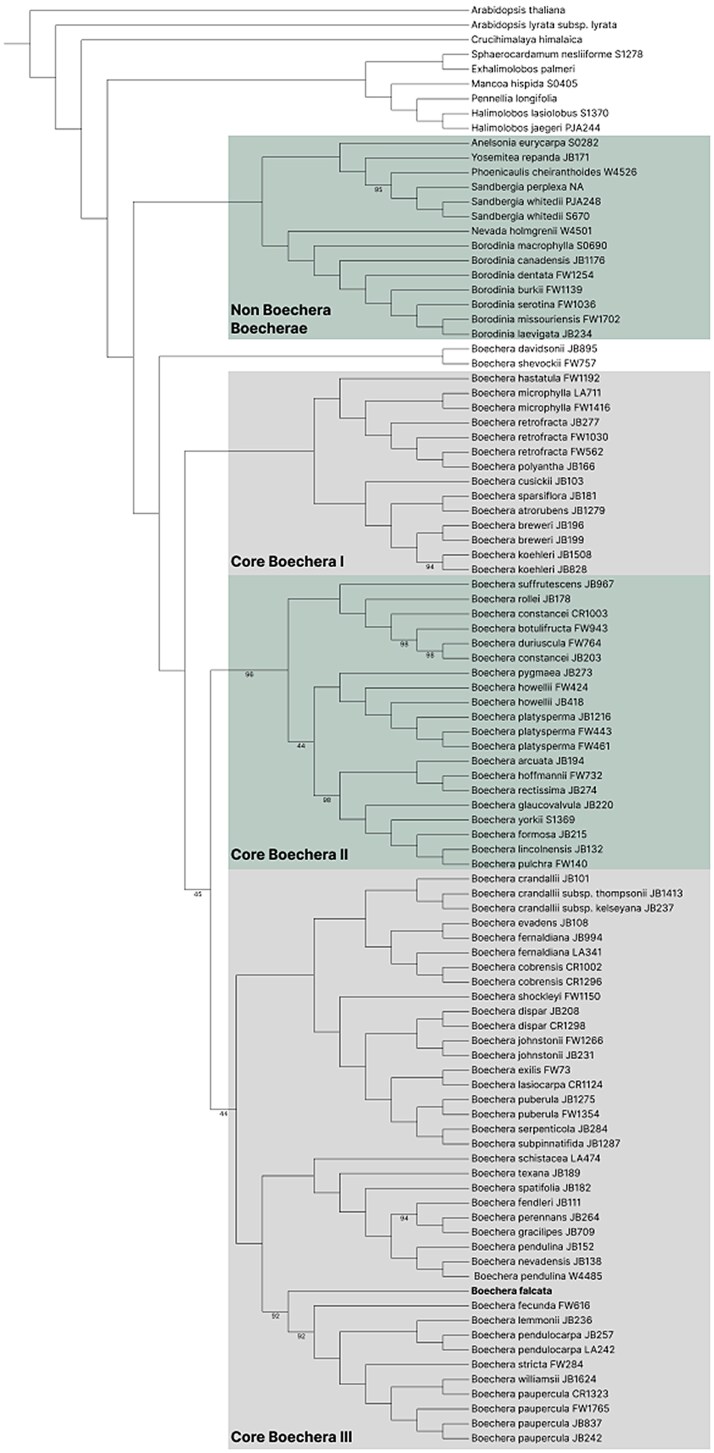
Phylogenetic tree of Boechereae and related species based on combined Angiosperms353 and Brassicaceae764 probe sets. The tree is rooted with outgroup species including *A. thaliana* and *Schrenkiella parvula*. Major clades are highlighted. *Boechera falcata* is indicated in bold. Numbers at nodes represent bootstrap support values <100.

The combination of Angiosperms353 and Brassicaceae764 probe sets revealed a comprehensive evolutionary relationship within the Boechereae tribe. The tree strongly supports the deep divergence between *Boechera* sensu stricto and the non-*Boechera* Boechereae (NBB) clade, consistent with previous studies (Hay *et al*. 2023). Within *Boechera* s.s., we observed 3 main core groups (Core *Boechera* I, II, and III). *B. falcata*, the focus of our study, is positioned within the Core *Boechera* III, sister to what Hay *et al*. (2023) called the Boreal clade (Fig. 9). This placement provides new insights into the phylogenetic relationships of *B. falcata* within the broader *Boechera* genus. The tree topology and strong bootstrap support of many nodes demonstrate the power of combining these probe sets for resolving relationships within this complex group.

## Conclusion

To conclude, we present a high-quality genome assembly of the only *Boechera* species native to Eurasia, (the Far Eastern *B. falcata*) to the chromosome level, as well as an assembly of chloroplast and mitochondrial DNA. The highly homozygous genome, the presence of only sex alleles of the *APOLLO* gene and previously obtained cytoembryological data suggest that this species reproduces only sexually. It likely came to the Eurasian continent from North America across the Bering Land Bridge during the Pleistocene glacial maxima. In terms of genome morphology, structure, and size, as well as chromosome arrangement and organelle DNA organization, this species is quite close to its North American sexual relatives from the Boechereae tribe. Using molecular probes, it was possible to confirm the placement of *B. falcata* within tribe Boechereae, namely in the Core *Boechera* III group. A phylogenetic tree constructed based on the chloroplast DNA confirmed the phylogenetic analysis using molecular probes. Within our limited sampling for the chloroplast DNA phylogeny, *B. falcata* appears related to North American *Boechera*. Finally, the availability of the genome of the only Eurasian *Boechera* species will help studies of the evolution and phylogeny of Brassicaceae species, as well as apomixis researchers to unravel the complex hybridization events that form *Boechera* apomicts.

## Supplementary Material

jkaf117_Supplementary_Data

## Data Availability

Sequencing data and genome assemblies are available through NCBI BioProject PRJNA1134229. Repeats and gene annotation for both *B. falcata* and *B. stricta* can be found on GitHub at https://github.com/zilov/boechera_falcata_article. Supplementary data attached to the article. Supplemental material available at G3 online.
