# Supplementary material for: Genome structure and molecular phylogeny of the only Eurasian Boechera species, Boechera falcata (Brassicaceae)
Source: G3 (Bethesda). 2025 Jun 20;15(7):jkaf117. doi: 10.1093/g3journal/jkaf117 (PMC12239615; doi:10.1093/g3journal/jkaf117)
Supplement: jkaf117_Supplementary_Data [file jkaf117_supplementary_data.docx]

**
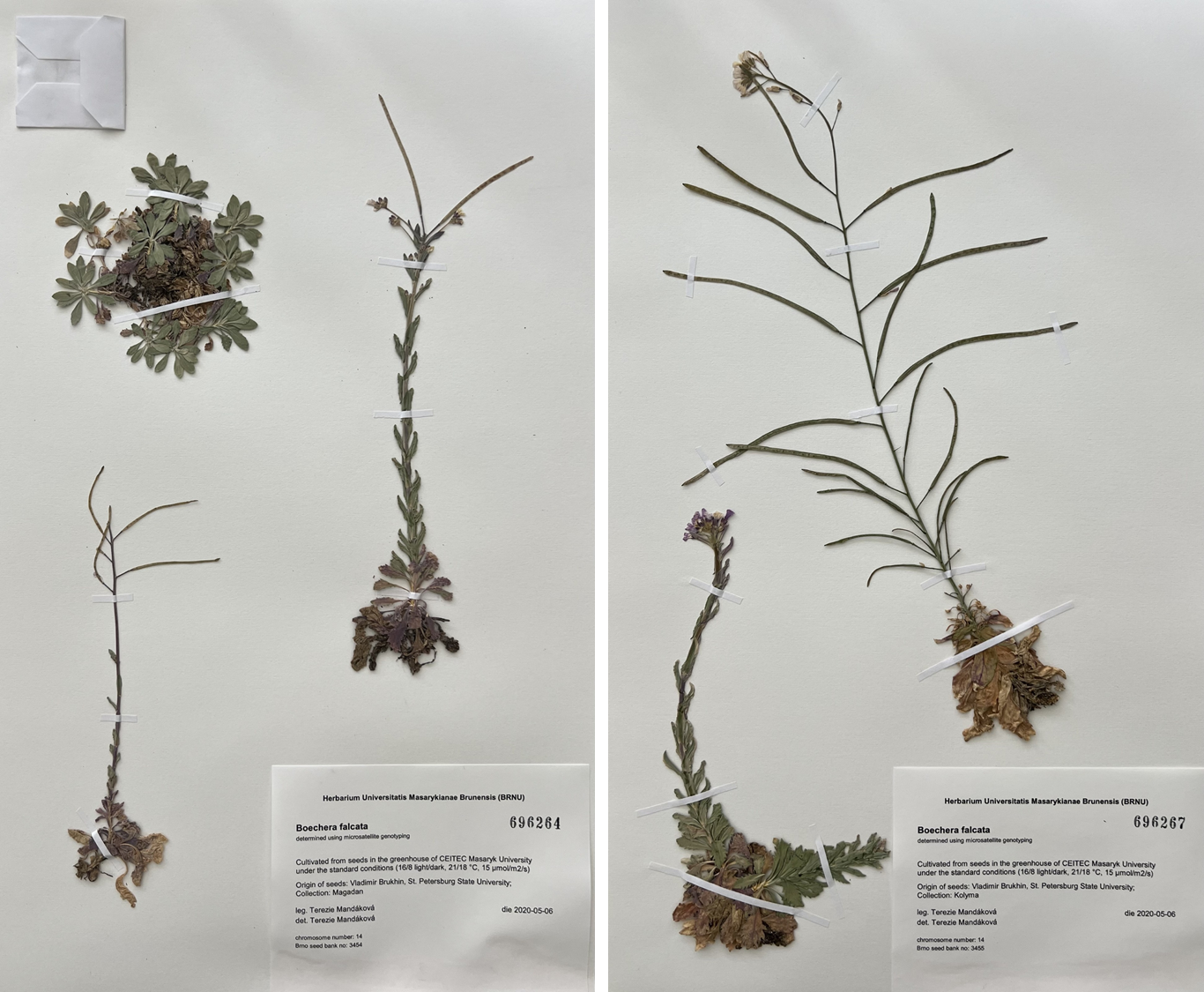
**

**Suppl. Figure 1** Herbarium specimen of *B. falcata* investigated, deposited in the herbarium of Masaryk University (BRNU).


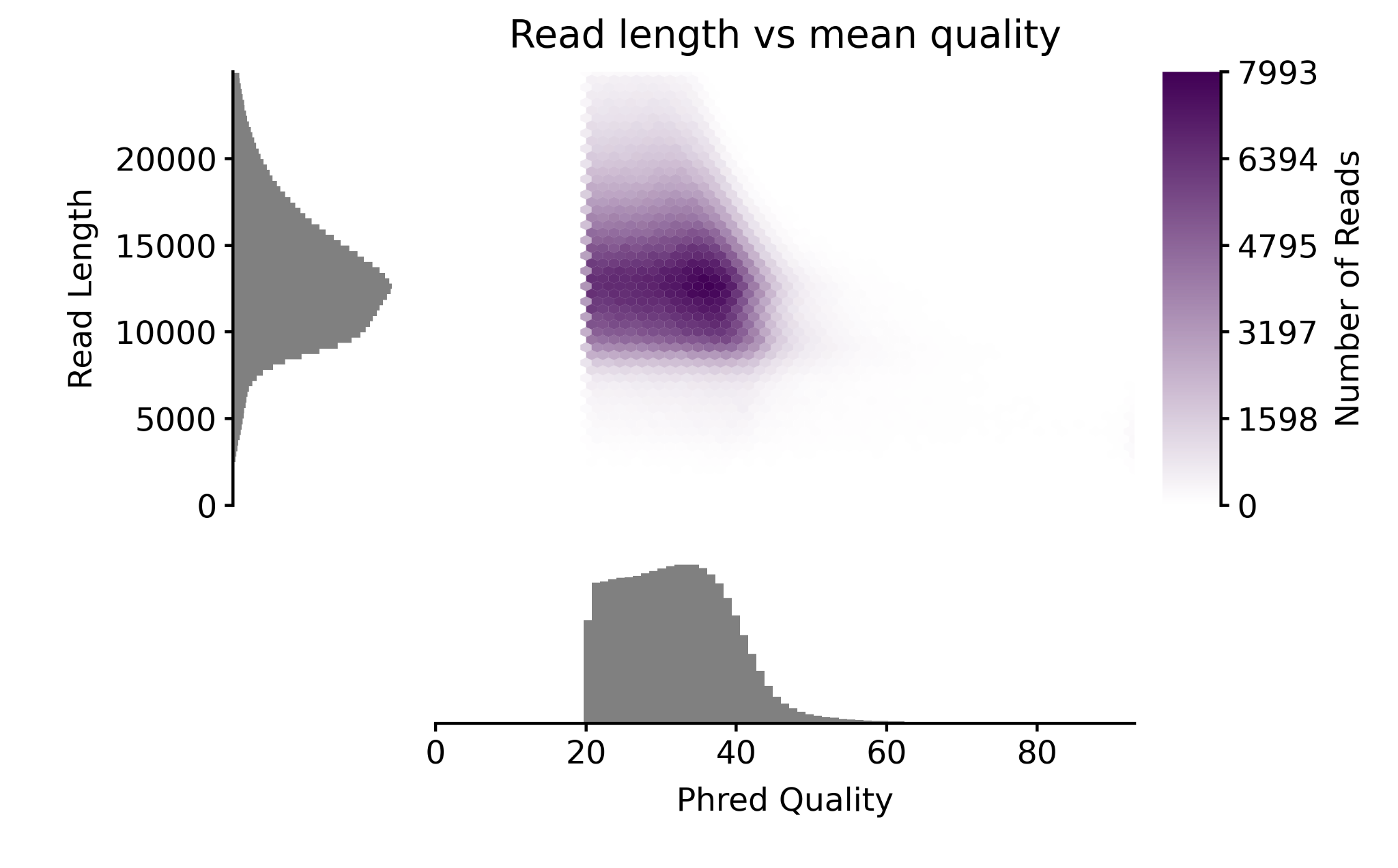


**Suppl. Figure 2.** Distribution of read length and phred quality scores for the HiFi library.


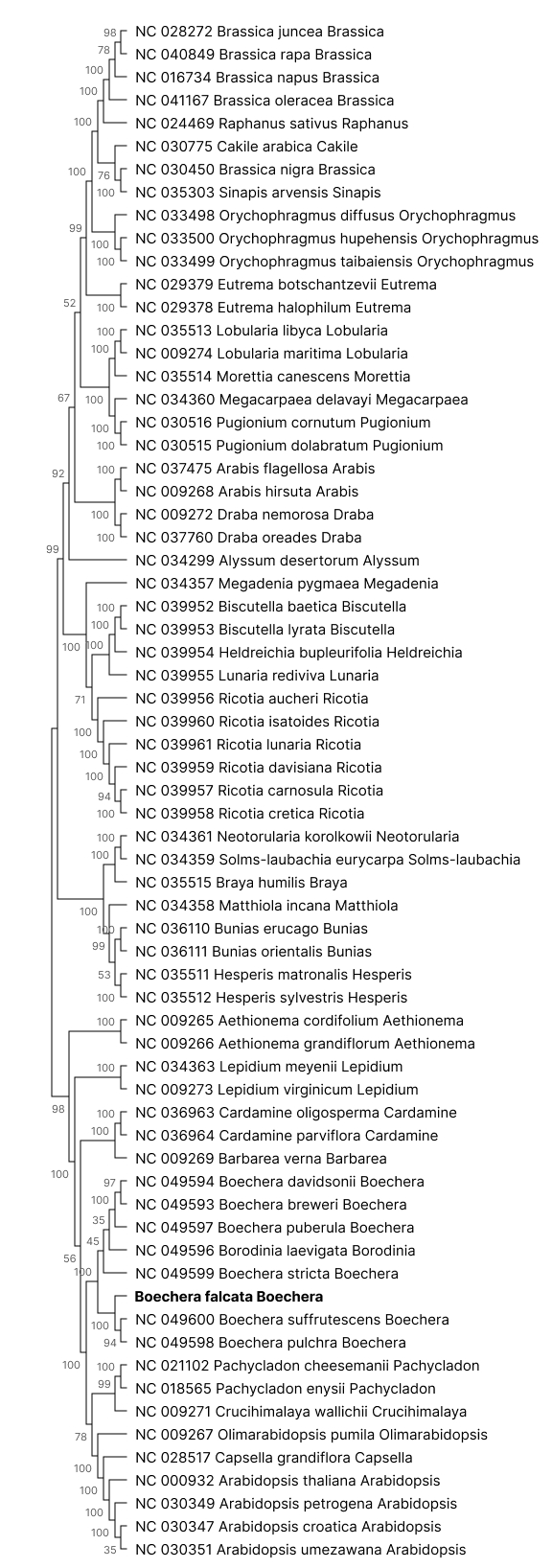


**Suppl. Figure 3.** Phylogenetic tree of Brassicaceae species based on the chloroplast genes.

**Suppl. Table 1.** Chloroplast genes used for phylogenetic analysis.

| Genes for chloroplast phylogenetic tree |
| --- |
| atpB |
| atpE |
| atpF |
| atpH |
| atpI |
| clpP |
| ndhA |
| ndhB |
| ndhC |
| ndhE |
| petA |
| petB |
| petD |
| petG |
| psaA |
| psaB |
| psaC |
| psaI |
| psbA |
| psbB |
| psbC |
| psbD |
| psbE |
| psbF |
| psbH |
| psbK |
| psbL |
| psbM |
| psbN |
| psbT |
| rbcL |
| rpl16 |
| rpl22 |
| rpl23 |
| rpl33 |
| rpl36 |
| rpoA |
| rpoB |
| rpoC1 |
| rpoC2 |
| rps18 |
| rps2 |
| rps3 |
| rps4 |
| rps7 |
| rps8 |
| ycf4 |
